# A high throughput proliferation and cytotoxicity assay for co-cultured isogenic cell lines

**DOI:** 10.1016/j.mex.2022.101927

**Published:** 2022-11-15

**Authors:** Syed Ahmad, Kris C. Wood, John E. Scott

**Affiliations:** a Department of Pharmaceutical Sciences, Biomanufacturing Research Institute and Technology Enterprise (BRITE), North Carolina Central University, Durham, NC, United States; b Department of Pharmacology and Cancer Biology, Duke University, Durham, NC, United States

**Keywords:** Cancer, PTEN, Isogenic

## Abstract

PTEN is a well-known tumor suppressor that is inactivated or suppressed at a high frequency in cancer. We sought an assay to screen compounds for ones that differentially inhibited proliferation or induced cytotoxicity in PTEN mutated cancer cells. We employed the isogenic pair of cell lines MCF10-A breast cell line (wild type, WT) and the same cell line with PTEN knocked out (KO) by CRISPR. We sought an assay where these PTEN WT and KO isogenic cell lines were co-cultured in the same well for compound testing. The KO cell line, but not the WT, was tagged with the red fluorescent protein mKate2. We employed a real time microscopic imaging instrument to identify cell populations in co-culture based on red fluorescence to obtain a cell count for each cell line. To acquire cytotoxicity data for each population, the dye CellTox Green was added to the media. To assess the assay, we determined the concentration response of paclitaxel. In order to assess the potential for screening, we performed mock screening in 384-well plate format. Thus, we developed a high throughput co-culture cell cytotoxicity and proliferation assay method that could be employed for any pair of cell lines to identify selective compounds.

## Graphical abstract

**Figure:**
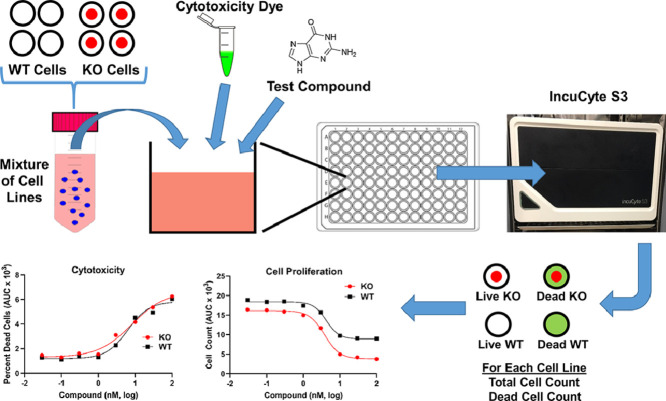

Specifications table

**Table:** 

Subject area:	Pharmacology, Toxicology and Pharmaceutical Science
More specific subject area:	Cell Biology and High throughput screening
Name of your protocol:	Proliferation and Cytotoxicity Assay for Co-Cultured Isogenic Cell Lines
Reagents/tools:	See Materials section
Experimental design:	Harvested isogenic PTEN cell lines in suspension were mixed together and then plated. The KO cell line expressed mKate2, a red fluorescent protein, while the WT cell line did not. Individual cells were identified using the Incucyte imaging instrument system and software. Identified cells were binned based on high or low red fluorescence, identifying the cells as KO or WT cells, respectively. This method of cell population counting was performed to measure cell proliferation of each cell line. The CellTox Green dye was added to the media to identify the dead cells (displaying high green fluorescence) and thus percent dead cells were separately calculated for each population.
Trial registration:	Not applicable
Ethics:	Not applicable
Value of the Protocol:	• The assay can measure proliferation and cell death in real time for each population in a co-culture of two cell lines • The method is amenable to high throughput screening for identifying selective compounds

**Description of protocol**

## Background and Rationale

PTEN is a well validated tumor suppressor protein that is a negative regulator for the PI3K/Akt signaling pathway [1,2]. Therapeutics that kill or inhibit the proliferation of cancer cells with mutated PTEN, but spare normal cells, may be clinically useful for treating PTEN mutated cancer. Synthetic lethal is a genetics term to describe the situation when two genes are mutated separately in different cells and have no impact on viability, but if both are mutated in the same cell, then the consequence is cell death. Applied to drugs, synthetic lethal is when a drug is cytotoxic to cells with a specific mutation or deficiency, but is not lethal to normal non-mutated cells [3].

One method to identify compounds that are selectively toxic for cells with a cancer-associated genetic alteration has been to employ isogenic cell lines where the cell lines are genetically identical, except for the mutation or deletion of a specific oncogene or tumor suppressor gene. High throughput screening (HTS) of compounds using isogenic cells mixed together before plating (co-cultured) provides potential advantages including a precise, simultaneous internal control for each compound tested and with high throughput speed. High throughput assays have been reported using isogenic cell lines in co-culture where the two cell lines are differentiated by tagging the cell lines with different fluorescent proteins [4,5]. These assays measure relative proliferation or cell counts of each population and are amenable for detecting compounds that differentially inhibit cell proliferation. However, one limitation of these assays is that cytotoxicity could not be directly measured in these assays. Thus, compounds that inhibit cell proliferation of both cell populations, but selectively induce cytotoxicity in the targeted cell population (a type of synthetic lethal hit) would be missed in a screen.

We sought to develop an assay to identify compounds and drugs that differentially inhibit the proliferation of PTEN mutated cells or kill PTEN- cells with a synthetic lethal like activity. Thus, we developed a high throughput, co-culture real time imaging assay that enabled quantification of proliferation and cytotoxicity in isogenic PTEN- and PTEN+ cell lines. This assay would enable the screening of compounds and drugs for ones that differentially inhibit the proliferation or induce cytotoxicity of the PTEN- cells. We employed the MCF-10A cell line which is a breast cell line that is immortalized, but not transformed. This cell line is often used in breast cancer research as a normal-like breast line. Thus, we used the parental MCF-10A (WT, PTEN+) and MCF-10A with homologous Clustered Regularly Interspaced Short Palindromic Repeats (CRISPR)-mutated PTEN genes (KO) such that its expression was eliminated. We sought an assay where the WT and KO cell lines could be mixed together in the same well for screening compounds. In order to differentiate the two cell populations, we transduced the KO cell line with mKate2, a RFP, while the WT cell line was transduced with empty vector. Individual cells were identified using the Incucyte imaging instrument system and software. Identified cells were binned based on high or low red fluorescence, identifying the cells as KO or WT cells, respectively. This method of cell population counting was performed to measure cell proliferation of each cell line. The CellTox Green dye is a membrane impermeant cytotoxicity dye that fluoresces green only when it binds DNA of cells that have lost membrane integrity i.e. dead cells. This dye was added to the media at the same time as compound or DMSO treatment to identify the dead cells (displaying high green fluorescence) and thus percent dead cells were separately calculated for each population. Thus, the dead WT cells were identified as ones that had low red fluorescence and high green fluorescence and the dead KO cells were identified as ones that had high red fluorescence and high green fluorescence.

The use of this assay method to identify synthetic lethal like drugs may have significant advantages. The internally-controlled nature of the assay may identify hits that would be missed with typical screening methods. The co-culture nature of the assay ensures that every well has an internal control (PTEN+) to select hits that have selective activity and thus reduce follow-up effort, false positives and triaging of compounds since most cytotoxic compounds are expected to be non-selective. Furthermore, the internally controlled nature of the assay may identify hits that would be missed if the compound activity is below a typical 50% cut-off. For example, compounds inducing 20% cytotoxicity against the PTEN- cells may be detected as a hit if the activity against the PTEN+ cells was only 5%. In this way, one could identify weakly active (but selective) compounds that would normally be missed in a traditional parallel/sequential screening approach or with assays that only detect cell proliferation. Additionally, the real time imaging aspect of the assay could allow the identification and characterization of selective hits based on kinetics of induction of cytotoxicity, i.e. slow kinetics for WT and rapid onset of activity for KO cells, where endpoint assays may not differentiate the two rates. The measurement of both cell growth and cytotoxicity may enable the detection a type of synthetic lethal compound/drug that is anti-proliferative to both PTEN- and PTEN+, but only cytotoxic to the PTEN- cells.

## Materials

Cell lines

• Human PTEN (-/-) MCF10A Cell Line (Horizon Discovery, cat# HD 101-006)
• MCF-10A cell line (Horizon Discovery)
• The PTEN KO cell line was transduced with a lentiviral construct expressing the red fluorescent protein (RFP) mKate2 (Capital Biosciences Inc., Gaithersburg, MD, cat. # VSL-0049, Lenti-mKate2P virus with puromycin selection marker). The PTEN WT cell line was transduced with the same vector from the same supplier, except it was an empty vector, i.e. without the mKate2 gene. The cell transduction and selection of cell lines with puromycin (10 µg/ml) was performed as reported previously [6].

Equipment

• IncuCyte S3 Live Cell Analysis System (Sartorius, Ann Arbor, MI)
• Water Jacket CO_2_ Incubator (ThermoFisher)
• Vi-Cell XR Cell Viability Analyzer (Beckman Coulter, Brea, CA)
• D300 Digital Dispenser with T8 Cassettes (Tecan, Morrisville, NC)
• Centrifuge (Beckman Coulter)
• Multidrop (ThermoFisher)
• Pintool head and automated liquid handling (Biomek NX, Beckman Coulter)

Reagents

• CellTox Green (Promega, Madison, WI, cat # G8731)
• Paclitaxel (Sigma-Aldrich, St. Louis, MO, cat# 33069-62-4)
• MycoAlert kit (Lonza, Basel, Switzerland)
• trypsin-EDTA (Corning, cat # 25-053-CI)
• DMEM/F12 (Hyclone, cat # SH30023.01)
• L-glutamine (Hyclone, cat # SH3003401)
• HEPES (MP, # 1688449)
• horse serum (Hyclone, cat # SH30074.03)
• insulin (Gibco, cat # 12-585-014)
• EGF (Sigma, cat # E9644)
• Hydrocortisone (Stemcell cat # 07904)
• cholera toxin (Sigma-Aldrich, cat # 9012-63-9)
• penicillin/streptomycin solution (Gibco, cat # 15-140-122)
• puromycin (Acros, cat # 58-58-2)
• DMSO (Corning, cat # 25-950-CQC)
• Benzethonium (MP, cat # 221661)
• DPBS/MODIFIED (Hyclone, cat # SH30028.03)

Software

• IncuCyte Cell-by-Cell Analysis Software Module (Sartorius, Ann Arbor, MI)
• Prism 9 software (Graphpad)

Plasticware

• 96-well black walled, clear bottom plates (Corning, cat. #3603)
• 384-well black walled, clear bottom plates (Corning, cat. #3764)
• Corning T150ml flasks (cat # 10-126-34)
• 50 ml conical centrifuge tubes (Corning, cat # 4490)
• 15 ml conical centrifuge tubes (Corning, cat # 430790)
• 75 mL Flask (Corning, cat # 07-202-000)
• Microcentrifuge Tubes, Eppendorf, 0.6 mL (Fisher Scientific, cat # 02-681-311)
• Pipet Tips, Sterile, P1000 BIOTIX™ (Rainin, cat # 12-111-369)
• Pipet Tips, Sterile, P200 BIOTIX™ (Rainin cat # 12-111-367)
• Pipet Tips, Sterile, P20 BIOTIX™ (Rainin cat # 12-111-366)
• Pipette, Aspirating, 2 mL (Corning, cat # 357558)
• Pipette, Serological, 10 mL (Corning cat # 4488)
• Pipette, Serological, 1 mL (Corning cat # 356521)
• Pipette, Serological, 25 mL (Corning cat # 4489
• Pipette, Serological, 2 mL (Corning cat # 4486)
• Pipette, Serological, 5 mL (Corning cat # 4487)
• Reservoir, 50mL (Corning cat # 4871)

## Cell line growth conditions

Maintain cell lines at 37°C in a humidified incubator at 5% CO_2_.

Cell culture media:

 DMEM/F12 media supplemented with
 2.5 mM L-glutamine
 15 mM HEPES
 5% horse serum
 10 µg/mL insulin
 20 ng/mL EGF
 0.5 µg/ml hydrocortisone
 0.1 µg/ml cholera toxin
 100 U/ml penicillin
 100 U/ml streptomycin.
 10 µg/ml puromycin (only for propagation of transduced cell lines)

After all supplements are added, filter the complete media using a 500ml filter system (Corning cat# 09-761-102).

Puromycin is used in media for propagation of the transduced cell lines, but not in the media used for the assay.

After receipt from the vendor and within two weeks of initial culturing, the cell lines should be amplified and frozen within 2-3 passages from original cells. We recommend that all experiments be performed with cells that are <20 passages from the original cell culture.

## Protocol

### Cell Harvesting

1. Seed a T-150 flask for the WT cell line and another flask for the KO cell line and culture in complete culture media (“media”) until cells reach 80-90% confluency.
2. Warm media, PBS and Trypsin-EDTA solution cell dissociation solution to 37°C.
3. Remove and discard media and wash cell monolayers twice with 10 ml of warm PBS.
4. Add 5 ml of warm cell dissociation solution to the flasks and incubate at 37°C for about 3-5 minutes. Gently tap the flasks to dissociate the cells and confirm dissociation using the microscope.
5. Add 10 ml of fresh media without puromycin and withdraw suspended cells from the flasks and transfer to a 50 ml conical tube.

Note: All subsequent steps that that use the term “media” are referring to complete media as above, but without puromycin.

6. Centrifuge tubes at 180 x g for 5 min at room temperature
7. Remove the supernatant without disturbing the pellet and resuspend the cell pellet in 10 ml of warm media with gentle vortexing
8. Take 500 µL of suspended cells and count cells using the Vi-Cell XR Cell Viability Analyzer according to manufacturer's instructions and using trypan blue packs for the instrument

### 96-well plate protocol

1. Adjust both cell suspensions to 100,000 cells/ml by adding warm media
2. Mix PTEN KO and WT at 1:1 ratio in culture media by mixing equal volumes of the cell suspensions so that cells are 5,000 cells/50 µL and gently vortex
3. Dispense 50 µL of media in each well in a 96-well plate (Corning, Cat# 3603) by Multidrop. Subsequently, add 50 µL of mixed cell suspension to each well by Multidrop, bringing the assay volume to 100 µL per well (total 5,000 cells/well). Alternatively, this step can be done by hand with a pipettor.

Note: Addition of media first, followed by cells results in more even distribution of cells for imaging.

4. Allow plate to incubate in the biosafety hood for 40 minutes at room temperature

Note: This incubation step at room temp results in more even distribution of cells and improves imaging

5. Put plate into 37°C incubator overnight
6. Add CellTox Green dye to all wells at 1:1000 dilution (100 nl) of the stock solution provided by the manufacturer using the D300 Digital Dispenser and T8 cassettes, following D300 manufacturer's instructions. Alternatively, the dye could be diluted into media first and then manually added to the wells in larger volumes (e.g. 10 µl/well) that result in the same final concentration of dye.
7. If compounds are tested in the assay: Add DMSO (solvent control) and test compound dissolved in DMSO to wells at desired concentration using the D300 instrument. Alternatively, the compound and DMSO could be diluted first into media and then added manually to wells in larger volumes (e.g. 10 µl/well) that result in the desired final concentration of compound and DMSO.

Note: All wells, including control wells, should be adjusted to the same final concentration of DMSO. When compounds are tested, use the D300 to directly deliver compounds dissolved in DMSO to create single point tests or 8-point half-log serial dilutions. Program D300 to adjust DMSO in all wells to the same final DMSO concentration of 0.1% of assay volume with T8 or T4 cassettes following the manufacture's procedure for the instrument.

Note: The CellTox Green dye solution is a DMSO solution. Therefore, the actual final DMSO in each well will be 0.2% DMSO – 0.1% from the dye and 0.1% from the compound/DMSO. We recommend to not exceed 0.2% final concentration of DMSO in the well unless cell tolerance to higher DMSO concentrations has been proven first.

8. Place the plate into the IncuCyte instrument that is located inside an incubator.
9. Monitor wells by phase contrast, red fluorescence and green fluorescence using the Cell-by-Cell software using the following steps.
  • Schedule plate imaging by clicking schedule to acquire using IncuCyte software 2019B rev2
  • Click the + sign, select scan on schedule, click next.
  • Create a new vessel by clicking NEW, click next.
  • Select standard for scan type, click next
  • In scan settings, select adherent Cell-by-Cell, Phase, red and green channel and 10X lens objective, click next
  • Select vessel Corning Cat # 3603, click next
  • Specify the location in the drawer for the vessel, click next.
  • Specify the scan pattern to use for image acquisition, selects wells to be scanned and five images per well, click next.
  • Provide information about the vessel/s, name, cell type, date to identify your vessel, click next.
  • Define an analysis to launch automatically after vessel has been scanned, or defer analysis until later, click next.
  • Create a new schedule with scans at interval of 6 hours and select stop scanning 3 -4 days after first scan. Make sure first scan is at least 40 minutes after placing vessel in the IncuCyte instrument, and 40 minutes of gap with next scan vessel. Click next.
  • Verify information is correct before continuing, if everything is correct add vessel for schedule
  • After scanning is complete, analyze data using IncuCyte software and Graphpad Prism as outlined below.

Note: We recommend scheduling the total plate read time for 24 hr more than is desired in case post-experiment data analysis indicates longer incubation times would be informative.

### Semi-automated high throughput 384-well plate screening protocol

1. Adjust both cell suspensions to 36,000 cells/ml by adding warm media
2. Mix PTEN KO and WT at 1:1 ratio in culture media by mixing equal volumes of the cell suspensions so that cells are 900 cells/25 µL and gently vortex
3. Dispense 25 µL of media in each well in a 384-well plate (Corning, Cat# 3764) by Multidrop. Subsequently, add 25 µL of mixed cell suspension to each well by Multidrop, bringing the assay volume to 50 µL per well (total 900 cells/well).

Note: Addition of media first, followed by cells, results in more even distribution of cells for imaging.

Note: We recommend 900 cells/well. Lower numbers gave greater variability.

4. Allow plate to incubate in the biosafety hood for 40 minutes at room temperature

Note: This incubation step at room temp results in more even distribution of cells and improves imaging

5. Put plate into 37°C incubator overnight
6. Add CellTox Green dye to all wells at 1:1000 dilution (50 nl) of the stock solution provided by the manufacturer using the D300 Digital Dispenser and T8 cassettes, following D300 manufacturer's instructions.
7. Transfer 50 nl/well DMSO (0.1% final) and/or test compound dissolved in DMSO to wells at desired concentration using a pintool head on a Beckman NX instrument. The transfer is from a DMSO or compound plate to the assay plate. Alternatively, compounds could be delivered by D300 for a small number of compounds.

Note: All wells, including control wells, should be adjusted to the same final concentration of DMSO. When compounds are tested, use the D300 to directly deliver compounds dissolved in DMSO to create single point tests or 8-point half-log serial dilutions. Program D300 to adjust DMSO in all wells to the same final DMSO concentration of 0.1% of assay volume with T8 or T4 cassettes following the manufacture's procedure for the instrument.

Note: The CellTox Green dye solution is a DMSO solution. Therefore, the actual final DMSO in each well will be 0.2% DMSO – 0.1% from the dye and 0.1% from the compound/DMSO. We recommend to not exceed 0.2% final concentration of DMSO in the well unless cell tolerance to higher DMSO concentrations has been proven first.

8. Place the plate into the IncuCyte instrument that is located inside an incubator.
9. Monitor wells by phase contrast, red fluorescence and green fluorescence using the Cell-by-Cell software using the steps listed above.

### Post-experiment image analysis

1. In order to bin the identified cells (using Cell-by-Cell analysis software) into WT (low red fluorescence) and KO cells (high red fluorescence), set the gate to differentiate high red fluorescence (KO) from low red fluorescence (WT). This gate should be set based on the fluorescence of representative low and high red fluorescent cells. The lower limit for designating a red cell should be slightly above the maximal signal from representative low red fluorescent cells and the upper limit slightly above the highest observed red fluorescence intensity. This range between the lower and upper limits of red fluorescence values defines the KO (high red fluorescence) cells and below this range is the WT (low red fluorescence).
2. In order to determine cell death, set gate to differentiate high green fluorescence (dead) from low green fluorescence (live). This gate should be set based on the fluorescence of representative viable and dead cells in the images. The minimum fluorescence limit for dead cell designation should be set above the maximal viable cell fluorescence, while the maximum limit for fluorescence should be set as slightly above the highest observed dead cell intensity. Hence, this signal range based on minimum and maximum fluorescence values defines cells that are dead and avoids spurious green fluorescence not coming from cells.
3. Use Incucyte software to calculate percent dead for each cell population. For WT, it would be the number of high green/low red cells divided by the total number of low red cells multiplied by 100. For KO, it would be the number of high green/high red cells divided by the total number of high red cells multiplied by 100.

### Data analysis by area under curve method

We recommend this data analysis method for small scale 96-well experiments.

1. For each time point, export cell numbers for each cell population for each well and the % dead for each cell population for each well into Excel spreadsheet
2. Import data into the Prism software and graph the data for each cell population on xy plots. The graphs should be cell count (per well) vs time (hr) for proliferation and dead cells (%) vs time (hr). A different line should be plotted for each test compound concentration.
3. Determine area under curve (AUC) using Prism software for each compound concentration
4. Plot cell count (AUC) vs compound concentration. Analyze by four parameter curve fit to get IC_50_ values for proliferation
5. Plot % dead cells (AUC) vs compound concentration. Analyze by four parameter curve fit to get EC_50_ values for cytotoxicity.

### Data analysis by end point method

Without an automated method to perform AUC calculations, we recommend the following method for analyzing high throughput data involving many wells/compounds.

1. Choose a time point to analyze, such as 48 or 72 hr
2. For just the chosen time, export cell numbers for each cell population for each well and the % dead for each cell population for each well into Excel spreadsheet
3. Determine relative KO cell proliferation: For each well (including control wells), divide KO cell count by the total cells counted (KO + WT cells). Subsequently, divide this fraction by the mean of the control well fractional values determined the same way, then multiple by 100. The resulting value is the relative KO cell count as percent of control.
4. Determine differential KO cytotoxicity: For each well, determine differential KO cytotoxicity by subtracting the % dead WT cells from % dead KO cells (% dead KO cells - % dead WT cells). The higher this value is, the more the treatment effected the KO cells over the WT cells.

### Hit identification for screening

1. For identifying differential cell proliferation inhibitor hits, we recommend using either a 50% inhibition cut-off for determining hits or use the z-score method, using mean and standard deviation of control wells, where hits would be those wells with z-score of ≤ -2.
2. For identifying differential cytotoxic hits, we recommend using an absolute cut-off such as ≥ 20% or use the z-score method and prioritize hits with highest z-score values.

## Protocol Validation

### Validation of cell counting for isogenic cell lines

**Fig. 1:**
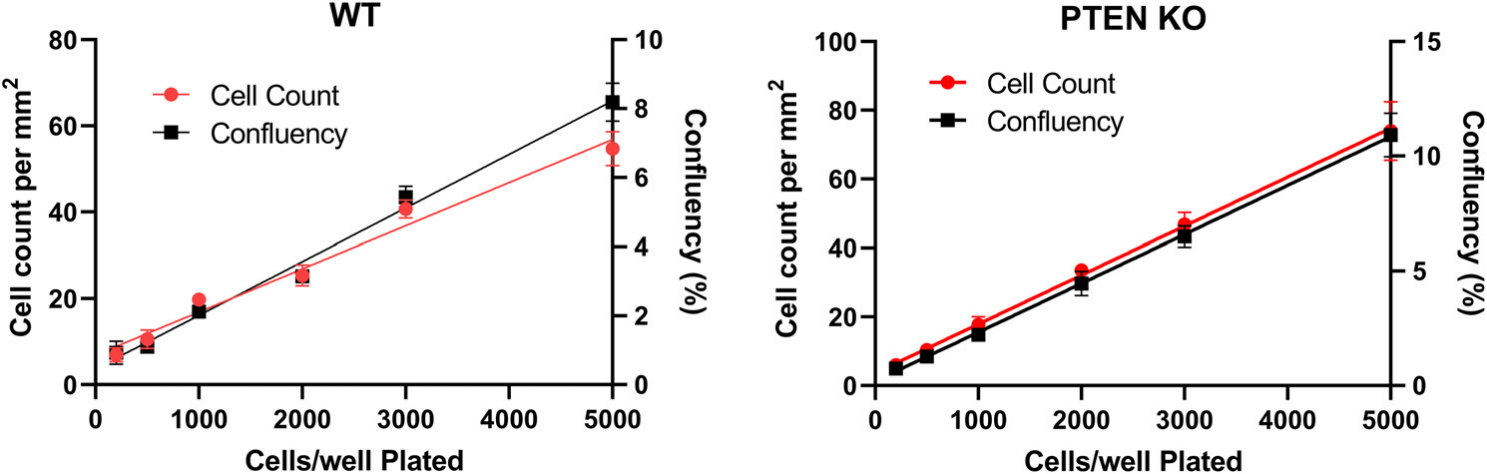
Validation of cell counting algorithm with confluency measurements. WT and PTEN KO MCF-10A cells were separately plated at the indicated number of cells per well. After overnight incubation, the plates were imaged in the IncuCyte instrument. The instrument software generated cell count and cell confluency (as indicated) for each well. Graphs are representative of N=3 independent experiments and the data points and error bars represent the mean ± SD of triplicate technical replicates.

This assay scheme depends on the identification of individual cells using phase contrast imaging. Thus, we sought to validate the Incucyte software's (Cell-by-Cell^TM^) ability to identify and count cells using phase contrast by comparing data generated by robust confluency determinations with the same instrument. We titrated the number of cells per well as pure cell lines (not mixed) and measured both confluency and cell counts (Fig. 1). We observed a close association between the cell count and confluency measurements. These data indicated that cell identification by the software was valid for these cell lines.

### Selective counting of PTEN KO and WT cells in co-culture and cytotoxicity dye tolerability

**Fig. 2:**
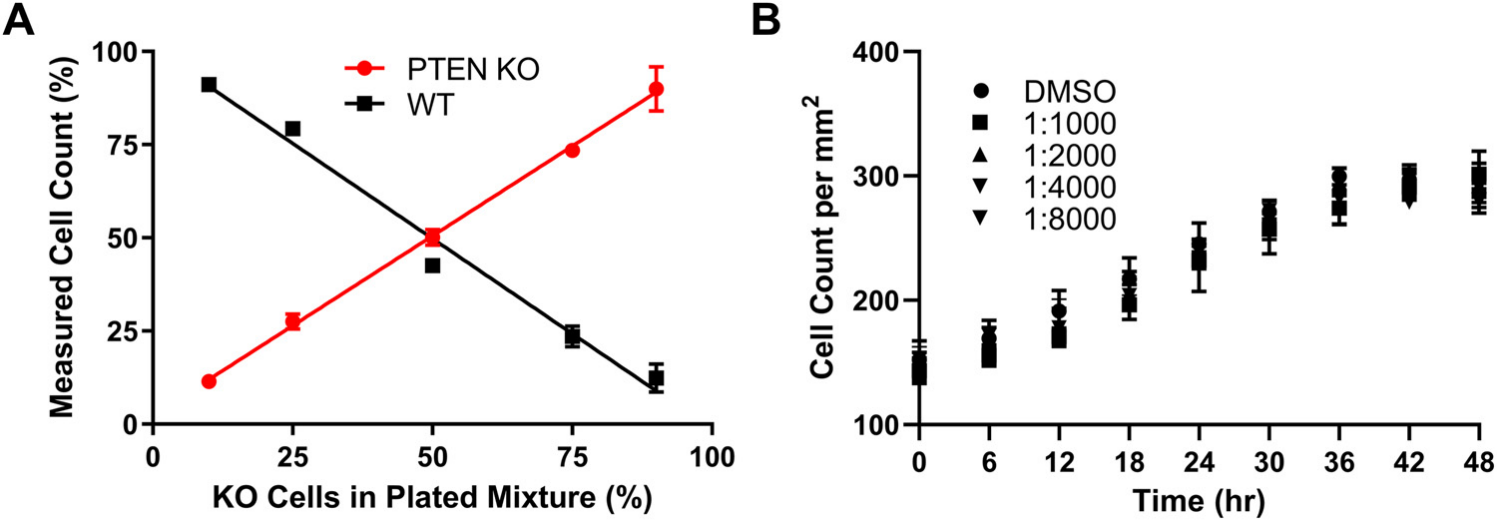
Cell count determination of two different cell populations in co-culture and tolerability to cytotoxicity indicator dye. (A) WT and KO cells were harvested and single cell suspensions mixed together to achieve the indicated percentages of KO cells, with the remaining percentage composed of WT cells, and then these mixtures were plated. After 18 hr, the wells were imaged in the IncuCyte instrument and the cell counts of KO (RFP+, high red fluorescence) and WT (low red fluorescence) cells were determined and percentages plotted as indicated. Data are representative of N=3 independent experiments and the data points and error bars represent the mean ± SD of triplicate technical replicates. (B) MCF-10A cells were treated with solvent (DMSO) or 1:1000 to 1:8000 dilution of the cytotoxicity dye stock solution provided by manufacturer and the cell count determined over time using the Incucyte instrument. Data are representative of N=2 independent experiments and the data points and error bars represent the mean ± SD of triplicate technical replicates.

We sought to determine if we could identify and count the two cell populations when they are mixed together for co-culture in the well. We generated changes in each cell population by mixing the KO and WT cells at different ratios (e.g. 10% WT, 90% KO) before plating and the next day the plate was imaged and processed to determine measured cell counts for each population (Fig. 2A). There was a direct relationship between mixed ratios and measured cell counts by percent of each population. These data verified that we could identify cells as WT or KO by low or high red fluorescence in co-culture.

In order to assess the tolerability of the cytotoxicity dye, we treated co-cultured cells with different dilutions of the dye stock solution (1:1000 to 1:8000) and measured total cell count over time (Fig. 2B). These data showed no impact on cell growth at any concentration of dye, so we used the manufacturer recommended concentration of 1:1000 dilution.

### Determination of paclitaxel concentration response in the co-culture assay

**Fig. 3:**
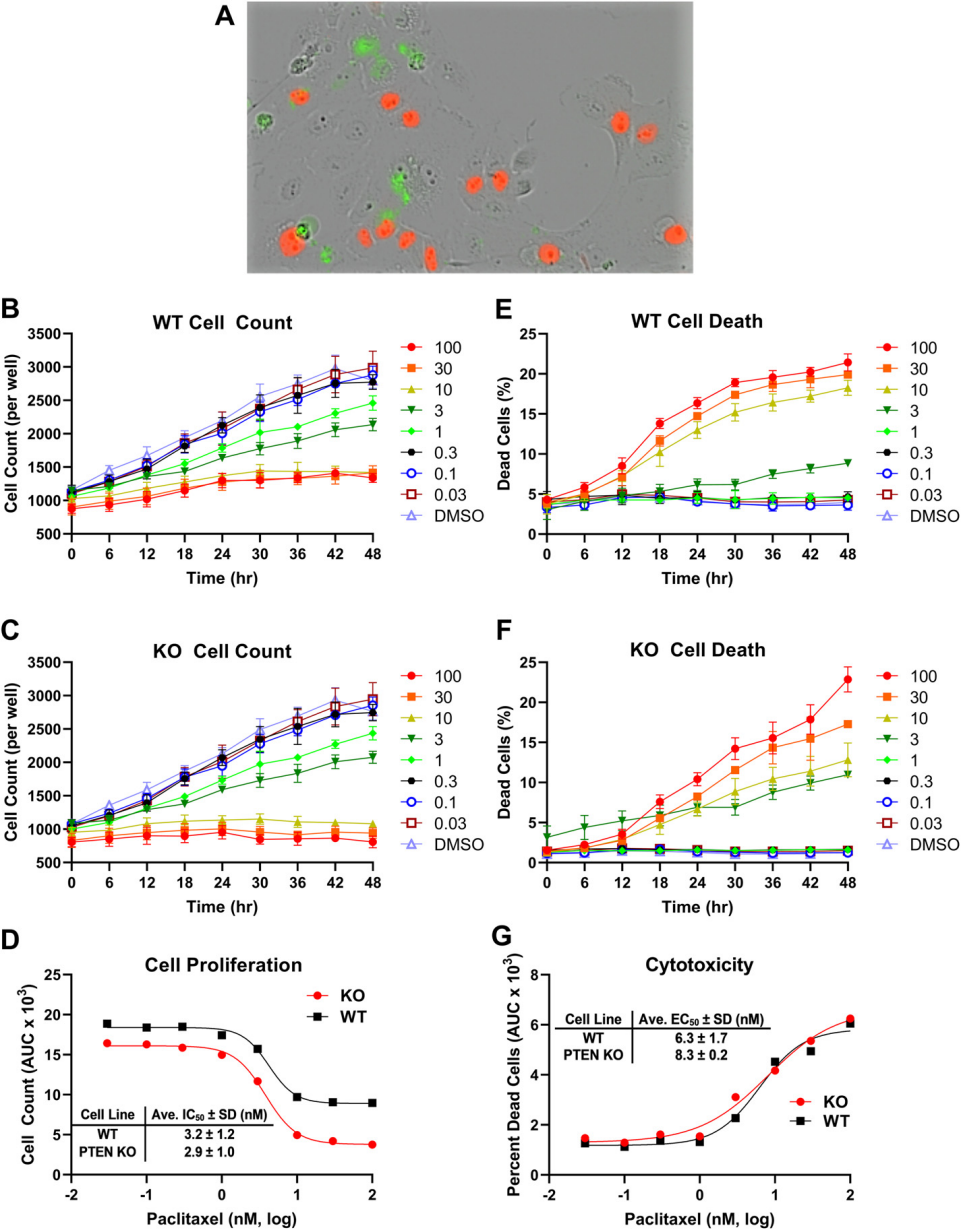
Determination of the concentration response of paclitaxel using the WT/PTEN KO co-culture proliferation and cytotoxicity assay. Co-cultured WT and PTEN KO cells were treated with various concentrations of paclitaxel (nM) or DMSO (solvent control) as indicated for 48 hr and cell proliferation and cytotoxicity was assessed. (A) A representative image was derived by merging phase contrast, red fluorescence (PTEN KO cells), and green fluorescence (dead cells) images for a plate well treated with 100 nM paclitaxel. (B,C) The kinetic data of cell count per well over time was plotted for each of the indicated concentrations of paclitaxel (nM) or DMSO for both populations. (D) The AUC for each concentration of paclitaxel was determined from the cell count kinetic data (B,C) for the WT and KO populations. (E,F) The kinetic data of percent dead cells per well over time was plotted for each of the indicated concentrations of paclitaxel (nM) or DMSO for both populations. (G) The AUC for each concentration of paclitaxel was determined from the percent dead cells kinetic data (E,F) for the WT and KO populations. Data shown are representative of N=3 independent experiments and the data points and error bars represent the mean ± SD of triplicate technical replicates (error bars in D and G obscured by data points), except for the IC_50_ and EC_50_ values derived from AUC data which were the mean ± SD values from the N=3 independent experiments.

To assess the performance of the assay to measure the effects of drugs, we performed concentration response studies with paclitaxel (Fig. 3). Paclitaxel represents an anti-proliferative and cytotoxic compound that was not expected to have selective activity for PTEN and therefore, similar responses were expected by the two cell populations in the assay. The merged phase/red fluorescence/green fluorescence images of paclitaxel-treated cells indicated cell death occurring, consistent with the known activity of paclitaxel (Fig. 3A). The kinetic cell count data indicated a concentration dependent inhibition of proliferation for both cell lines (Fig. 3B,C). The AUC for the cell count kinetic data was plotted verses paclitaxel concentration and a dose response curve was separately generated for the WT and KO cells (Fig. 3D). The average (N=3) IC_50_ values and standard deviation (SD) were 3.2 ± 1.2 and 2.9 ± 1.0 for the WT and KO cell counts, respectively, which are in close agreement with each other, as would be expected. The kinetic data for percent dead cells in each population over time indicated paclitaxel was inducing cell death in both populations (Fig. 3E,F). The AUC for the cytotoxicity kinetic data was separately generated for the WT and KO cells and plotted verses paclitaxel concentration to generate concentration response curves (Fig. 3G). These data showed the potent cytotoxic nature of paclitaxel in both populations and average (N=3) EC_50_ values and SD were 6.3 ± 1.7 nM and 8.3 ± 0.2 nM for WT and KO cell populations, respectively.

### Co-culture proliferation and cytotoxicity assay performance under simulated screening conditions in 384-well format

**Fig. 4:**
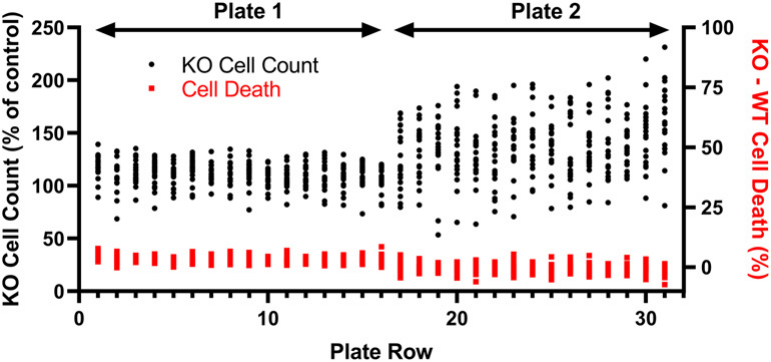
Co-culture proliferation and cytotoxicity assay performance in high throughput 384-well format. An equal mixture of WT and PTEN KO cells (750-900 total cells/well) were plated into 384-well plates. All wells were treated with DMSO (0.01% final) to simulate compound screening, with columns 1 and 2 serving as DMSO controls for calculations and benzethonium added to columns 23 and 24 as a qualitative cytotoxicity control (not plotted). Data shown were based on the 72 hr time point after DMSO addition for columns 3-22. The relative KO cell count (●), an indicator of cell proliferation, was determined as the fraction of KO cells to total cells counted per well normalized to DMSO control wells. Differential KO cell cytotoxicity (■) was determined by calculating percentage of counted KO cells that were dead (green) minus the percentage of counted WT cells that were dead. Plate 2, row P data was not plotted due to a liquid handling automation error. The data from two 384-well plates were plotted that represent N=2 independent experiments performed on different days.

The above data was generated in 96-well plates. We sought to generate proof-of-concept for using this assay in 384-well plate format which is commonly used for high throughput screening (HTS). Thus, we miniaturized the assay for 384-well plates and performed screening validation experiments consisting of delivering DMSO (instead of test compounds) to all the wells of 384-well plates in semi-automated fashion that simulates a high throughput screen of compounds. This type of DMSO-only testing is often used to determine an assay's suitability before screening actual drugs/compounds. We also treated columns 23 and 24 with benzethonium, a cytotoxic compound, as a qualitative control to verify detection of cytotoxicity. Columns 1-2 were used as DMSO control wells, as in a typical compound screen. Thus, there should be no wells displaying differential anti-proliferative or cytotoxicity in columns 3-22 since all wells are treated the same – just DMSO. Benzethonium has a detergent-like structure and at the 10 µM concentration used in these experiments, the benzethonium completely destroyed cell structure beyond recognition, leaving only a few intact cells. Thus, the cytotoxicity control wells did not provide valid and reliable data. In retrospect, a better cytotoxicity control to use in screening would be paclitaxel (or a cytotoxic compound like it) which is less cell structure destructive and more relevant since it is an oncology drug. There are many ways one could analyze this data. Without an automated way to calculate AUC, we chose to use the cell counts and cytotoxicity data from the 72 hr time point to generate proof-of-concept data. We first calculated the fraction of KO cell count to total cells counted for each well then normalized that value to the average fraction from the DMSO control wells (column 1-2) to derive percent of control values for each test well (column 3 – 22) (Fig. 4). There were signs of significant edge effects for the outer wells of the plate where cell proliferation was reduced. This could be improved by use of a plate seal and would likely improve the performance and variability of the assay. Despite the edge effects, there were no wells with ≤50% of KO cells compared to WT. If one used a typical 50% cut-off for hits, then no “hits” were obtained in this DMSO screen. If one used the z-score method of determining differential hits with a cut-off of ≤-2 z-score, there were also no differential hits. An analysis for differential cytotoxicity was also performed using the 72 hr data where the % dead for each population was calculated using the instrument software. This data was plotted as the difference in cytotoxicity between populations (% dead KO cells – % dead WT cells) and thus, a greater number represents a greater differential killing of KO cells (Fig. 4). The average and SD of this differential value was 1.4 ± 2.5 percentage points and all differential values were <9 percentage points. Thus, this assay and data calculation method resulted in a robust method to screen for cytotoxic compounds with differential ability to kill KO cells. Thus, by performing this type of analysis for both differential anti-proliferative activity and differential cytotoxicity, two types of hits may be extracted from screening data. These DMSO-only experiments indicated that the co-culture assay is robust enough for screening, with the cytotoxicity measurement producing particularly low background.

Once preliminary single-point hits from a screen are identified (which would be expected to occur at very low frequency), one could further verify the hit wells by a manual review of the kinetic data, raw cell count data and images before confirmation testing. This extra manual analysis could eliminate non-selective hits that are so highly cytotoxic that they destroy cell structure and thus cell count and cell death calculations are only based on a few intact cells, resulting in misleading values after data is calculated. Alternatively, one could set an automatic cell count cut-off for WT cells where potential hits with less than some absolute number of counted WT cells (based on controls) would be eliminated. One potential improvement on this assay could be the use of an imaging instrument capable of detecting three fluorescence colors and the WT cell line tagged with a different fluorescent protein that can be detected in multiplex with the RFP and the green cytotoxicity dye.

In sum, we developed a high throughput co-culture assay with isogenic cell lines to identify compounds that have differential anti-proliferative or cytotoxic activity against PTEN- cells. We demonstrated the identification of cell populations based on expression or lack of expression of a RFP and cytotoxicity determination with the CellTox Green cytotoxicity dye. This kinetic assay is amenable to screening collections of approved drugs and diverse compounds for synthetic lethal cytotoxicity for PTEN mutated cell lines. This high throughput screening approach could be used with any viable isogenic pair of cell lines (or any two cell lines) to identify selective compounds or drugs.

## CRediT authorship contribution statement

**Syed Ahmad:** Investigation, Methodology, Validation, Writing – original draft. **Kris C. Wood:** Conceptualization, Funding acquisition, Writing – review & editing. **John E. Scott:** Conceptualization, Funding acquisition, Methodology, Writing – original draft, Supervision.

## Declaration of Competing Interest

The authors declare the following financial interests/personal relationships which may be considered as potential competing interests:

John E. Scott and Kris Wood received financial support from the National Institutes of Health. K.C.W. is a founder, consultant, and equity holder at Tavros Therapeutics and Celldom and has performed consulting work for Guidepoint Global, Bantam Pharmaceuticals, and Apple Tree Partners.

## Data Availability

Data will be made available on request.
